# Analysis of the first *Taraxacum kok-saghyz* transcriptome reveals potential rubber yield related SNPs

**DOI:** 10.1038/s41598-017-09034-2

**Published:** 2017-08-30

**Authors:** Zinan Luo, Brian J. Iaffaldano, Xiaofeng Zhuang, Jonathan Fresnedo-Ramírez, Katrina Cornish

**Affiliations:** The Ohio State University, Department of Horticulture and Crop Science, Wooster OH, 44691 USA

## Abstract

*Taraxacum kok-saghyz* (TK) is a potential alternative crop for natural rubber (NR) production, due to its high molecular weight rubber, short breeding cycle, and diverse environmental adaptation. However, improvements in rubber yield and agronomically relevant traits are still required before it can become a commercially-viable crop. An RNA-Seq based transcriptome was developed from a pool of roots from genotypes with high and low rubber yield. A total of 55,532 transcripts with lengths over 200 bp were *de novo* assembled. As many as 472 transcripts were significantly homologous to 49 out of 50 known plant putative rubber biosynthesis related genes. 158 transcripts were significantly differentially expressed between high rubber and low rubber genotypes. 21,036 SNPs were different in high and low rubber TK genotypes. Among these, 50 SNPs were found within 39 transcripts highly homologous to 49 publically-searched rubber biosynthesis related genes. 117 SNPs were located within 36 of the differentially expressed gene sequences. This comprehensive TK transcriptomic reference, and large set of SNPs including putative exonic markers associated with rubber related gene homologues and differentially expressed genes, provides a solid foundation for further genetic dissection of rubber related traits, comparative genomics and marker-assisted selection for the breeding of TK.

## Introduction

Natural rubber (NR) is a biopolymer consisting of many isoprene units (C_5_H_8_)_n_ linked together in a *cis*-configuration^1^. NR is a critical global material because its high-performance properties cannot be matched by petroleum-derived synthetic rubbers in many applications requiring resilience, impact resistance, abrasion, and heat dispersion, among other desirable properties^2^. Furthermore, there is an imperative to replace petroleum-derived products with renewable natural resources^2^. In the United States, virtually all NR, harvested from *Hevea brasiliensis*, is imported from Southeast Asian countries such as Indonesia, Thailand and Malaysia, as well as African countries such as Côte d’Ivoire, Liberia and Cameroon. These countries provide over 96% of the world’s >12 megatonnes (mt) NR/year^3^.

However, the production of NR faces several serious threats. The rapid economic development of countries such as China and India has led to increasing demand and increasingly unstable prices for NR^2^. Moreover, since rubber harvesting is labor intensive, several countries, such as Malaysia, Thailand and Indonesia, have invested in lower input crops. In only four decades, palm oil plantations in Malaysia increased from 54,000 hectares (1960) to 4.05 million hectares (2005), as it displaced rubber tree acreage^4^, leading to a similar amount of deforestation in poorer countries in order to plant replacement *Hevea* trees. Other than the potential shortage of NR due to the increasing demand (6.8%/yr)^5^ and changes in land use, South American Leaf Blight (SALB), a fatal disease of *Hevea* caused by the fungal pathogen *Microcyclus ulei*, also poses a serious threat to NR production, the tire industry and the global economy^6^. SALB severely limits NR production in its native South America and would devastate South East Asian production if it established there. White Root Rot, caused by the fungal pathogen, *Rigidoporus lignosus*, has already spread throughout Southeast Asia and Africa and is currently the most common disease of *Hevea* in those regions^7^.

Many countries recognize the imperative to develop alternative sources of NR, and extensive research and development efforts are underway especially in the United States and Europe. *Taraxacum kok-saghyz* (TK), also called rubber dandelion, or rubber root, is an ideal rubber-producing crop because the quality of TK rubber is almost identical to *Hevea* rubber^8^ and it can be grown as a direct seeded annual crop. Its annual production system allows acreages of crop to be readily adjusted in response market needs^2^. Moreover, TK is adapted to broad temperate areas. However, wild TK performs poorly in normal cultivated fields and improvement of rubber yield and agronomic fitness through breeding efforts is essential. Despite this, only limited breeding efforts have been carried out since plants are usually self-incompatible, highly heterozygous, and rubber yield is only measurable at maturity^2^.

Crop domestication would be greatly accelerated by efficient application of developed genetic resources as are available in traditional crops such as corn and soybean. However, TK (2n = 16), with a total diploid genome size of 2.4 GB^9^, currently has limited publically available genetic resources. These are limited to 16,441 ESTs (GenBank: accession numbers DR398435 to DR403165 and GO660574 to GO672283), a linkage map^10^ and the chloroplast genome^11^.

In addition, some individual TK genes more or less related to rubber production have been identified. TK contains *cis*-prenyltransferases (CPTs), small rubber particle protein (SRPPs) and rubber elongation factor (REF), which share high sequence similarity with those present in *Hevea* ^12^. Three CPT homologues (*TkCPT1-3*) and five SRPP homologues (*TkSRPP1-5*) have been cloned and characterized in TK^12^. In addition, some receptors^13^ and activators^14^ have been implicated in rubber biosynthesis. Many other important rubber biosynthesis related genes, including all important rubber polymerase (rubber transferase (EC 2.5.1.20)) have yet to be identified. NR is synthesized from one priming allylic pyrophosphate (allylic-PP) molecule and the monomer isopentenyl pyrophosphate (IPP)^15^. The allylic-PP molecule, namely dimethyl allyl-PP (DMAPP), geranyl-PP (GPP) or *trans, trans*-farnesyl-PP (FPP), acts as an initiator in rubber polymerization, allowing subsequent sequential condensations of the non-allylic isopentenyl-PP (IPP) catalyzed by the rubber particle bound rubber transferase in the cytoplasm of plant laticifer cells^15^. The IPP precursors are synthesized either from the mevalonate (MVA) pathway in the cytosol or from the 1-deoxy-D-xylulose-5-phosphate/2-C-methyl-D-erythritol-4-phosphate (DOXP/MEP) pathway in the plastids, followed by the diffusion of IPP into the cytosol^15^. The MVA and MEP pathways are part of terpenoid backbone biosynthesis pathway. Rubber polymers (*cis*-1,4-polyisoprene) accumulate inside rubber particles that are surrounded by a contiguous monolayer membrane containing a species-specific complement of lipids and proteins, including rubber transferase^16^. Other than isoprenoid metabolism, Post *et al*. found a significant negative correlation between rubber production and the main storage carbohydrate, inulin^17^. When excess IPP accumulates, the HMGR enzyme—the key regulatory step for IPP biosynthesis—is inhibited^17^ and excess IPP is redirected to the downstream steroid biosynthesis pathway. When this relief pathway is also saturated, the accumulation of early rubber precursors (e.g. acetyl-CoA) cause a redirection of carbon flux upstream, and more inulin is synthesized. However, whether this redirection was a direct consequence of excess precursors and/or the increase in sterol levels or the consequence of pleiotropic effects has yet to be determined^17^.

Transcriptomic data is completely lacking in TK, but this information is instrumental to the identification of genes expressed during periods of active rubber production as well as determining genes directly related to rubber yield. Also, base changes in genes often lead to different expression or functionality. If polymorphisms occur in the genes involved in rubber biosynthesis, they may lead to changes in rubber concentration in TK roots. Hence, profiling transcriptomes of low-rubber and high-rubber-yielding TK genotypes would allow the identification of differentially expressed genes, potential genes related to rubber biosynthesis as well as any associated genetic polymorphisms. Such polymorphisms may be used to develop molecular markers for early screening of progeny, thereby accelerating breeding time, and to help dissect genetic features influencing rubber production in TK. RNA-Seq is an effective and efficient method for generating a reduced representation of a species’ genome, specifically targeting expressed genes^18^. RNA-Seq has become one of the major methods for SNP discovery and differential gene expression analysis, and has been used for transcriptome profiling in staple crops such as maize^19^ as well as the species with limited genomic resources in the Asteraceae family, such as *Stevia rebaudiana* (stevia)^20^ and *Carthamus tinctorius* (safflower)^21^.

In this study, we sequenced the first root transcriptome of TK focused on low and high rubber genotypes with the aim to identify TK homologues genes related to rubber yield and functionally annotate differentially expressed genes as well as detecting associated SNPs. The transcriptomic data developed here has also expanded and enriched the publically available TK root genomic resources, and may accelerate TK germplasm improvement.

## Results

### Transcriptome sequencing and *de novo* assembly

A total of 357,694,286 paired-end reads with lengths of greater than 100 bp were obtained from six TK accessions (GenBank accession number: TK6 (SRR5181667); TK9 (SRR5181665); TK10 (SRR5181664); TK14 (SRR5181663); TK18 (SRR5181662); TK21 (SRR5181661)). Before normalization, 141,000 transcripts representing 94,000 components were assembled. After normalization 10,779,087 reads were obtained for each orientation (7.8% of reads prior to normalization). *De novo* assembly using normalized data yielded 214,302 transcripts representing 71,581 components, from which 55,532 transcripts representing 19,514 components were finally produced after selection of transcripts with Transcript Per Millions (TPMs) values greater than one, using a pool of reads from all samples. The N50 statistic of the assembly using the non-normalized dataset (70GB) was 1460 bp, while the normalized subset (5GB) had an N50 of 1830bp. A comparison of Orthologue Hit Ratios (OHR) output a comparable number of transcripts between normalized and non-normalized data through the range of 20–100, although the number of transcripts was slightly larger in normalized than non-normalized data at higher OHR ranging from 60 to 100 (Figure S1).

### Sequence annotation and biological interpretation

Out of 55,532 transcripts, 42,316 (76.2%) had significant hits in the NCBI non-redundant protein (nr) database. The mean sequence length of transcripts with significant BLAST hits was 1602 bp [maximum (max) = 16,687, minimum (min) = 200, standard deviation (std) = 1018], while the mean sequence length of transcripts without significant BLAST hits was 691 bp (max = 4,630, min = 201, std = 475). Twenty-three point eight percent (23.8%) of the transcripts had no homologous sequences in the nr database, which could result from the presence of either untranslated mRNA, novel genes, genes unique to TK root, or assembly errors. Five species showing most frequent best local alignment for translation products of a nucleotide query sequence (BLASTx) hits to our transcriptome were *Vitis vinifera, Solanum tuberosum, Theobroma cacao, Solanum lycopersicum*, and *Prunus persica* (Figure S2).

#### Database Comparisons

**Figure 1:**
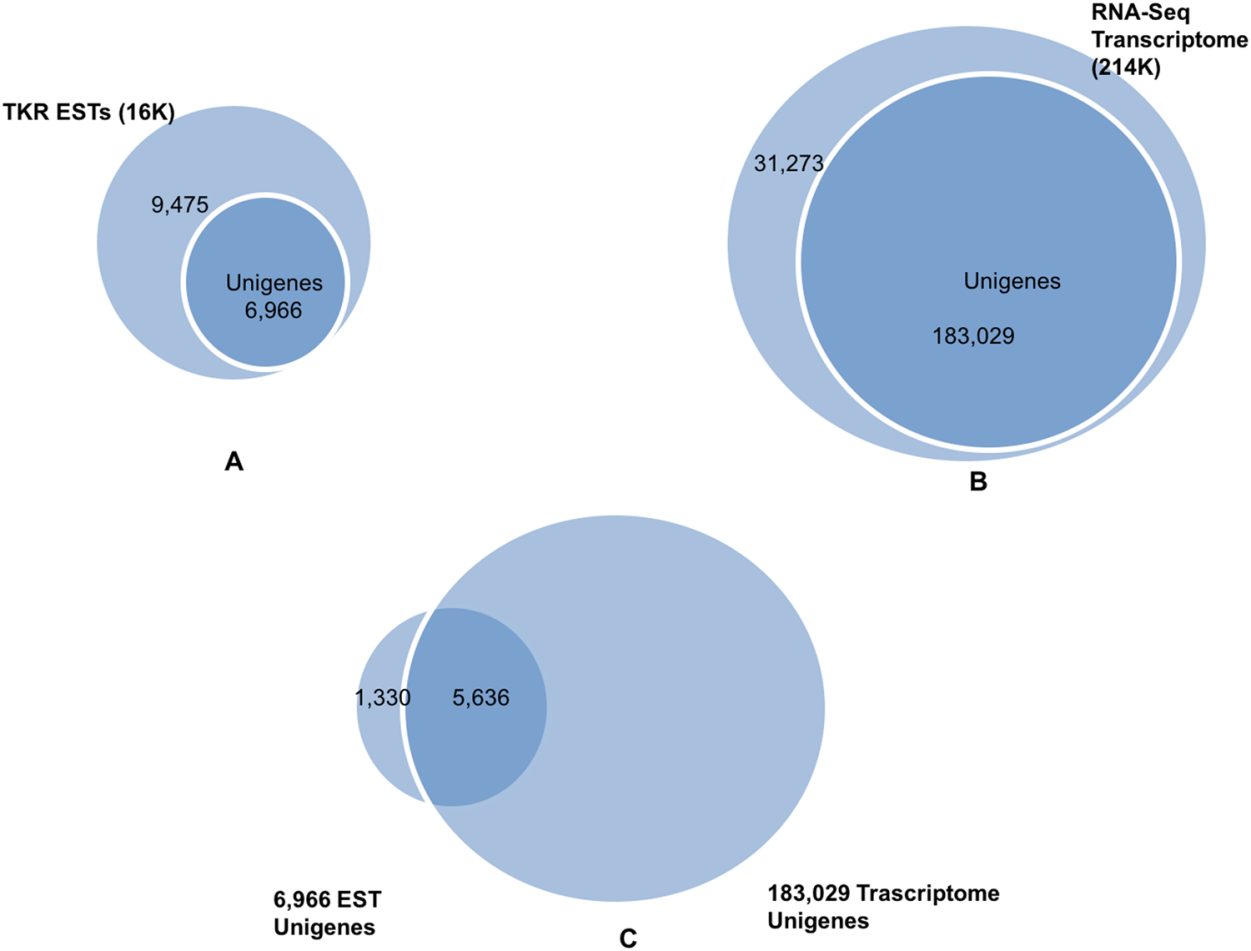
Unigene comparison between RNA-Seq transcriptome sequences and TKR ESTs. **(A**) The ratio of unigenes within the online TKR EST database (16 K); (**B**). The ratio of unigenes within RNA-Seq transcriptome dataset (214 K); (**C**). Reciprocal BLASTn search between the online TKR ESTs and RNA-Seq transcriptome dataset.

Comparisons were made between our raw transcriptome dataset (214 K) and an earlier online TK root (TKR) EST database (16 K). Assembly of the 16,441 public ESTs resulted in a unigene set of 6,966 transcripts and singletons (Fig. 1A). On the other hand, our transcriptome dataset generated 183,029 unigenes from 214,302 transcripts (Fig. 1B). The two sets of unigenes then were searched reciprocally using BLASTn with a cut-off e-value of 1e^−20^. A total of 5,636 (80.9%) EST unigenes from the original 16 K dataset were covered by our transcriptome dataset (Fig. 1C), but we also generated 170,638 (93.2%) unigenes that were not present in the TKR EST database (Accession No.: DR398435.1-DR403165.1; G0660574.1-G0672283.1). The N50 statistics were 744 bp and 1977bp for the EST unigenes and our transcriptomic unigenes, respectively.

#### Gene Ontology (GO) analysis

**Figure 2:**
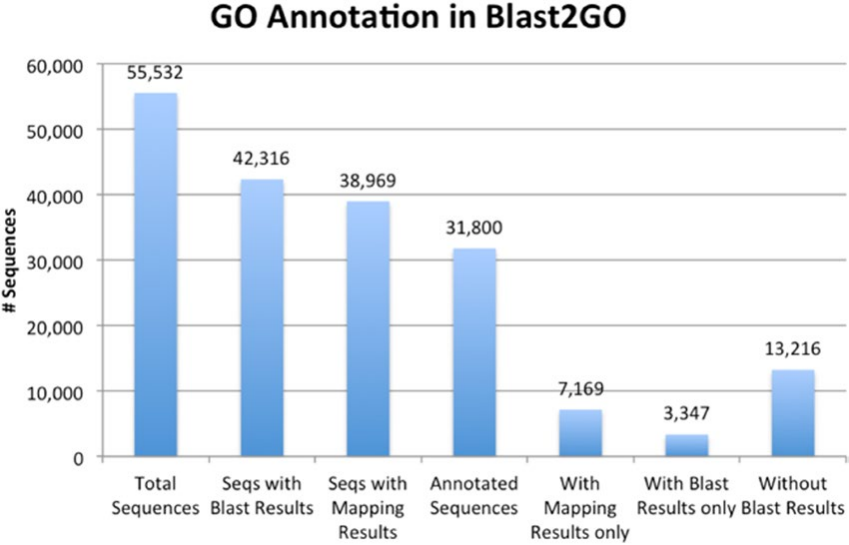
Gene Ontology (GO) annotation. Of 55,532 transcripts assembled, 42,316 transcripts show positive BLAST hits, leaving 13,216 transcripts without BLAST results. Of the 42,316 transcripts, 38,969 were assigned GO terms (sequences with mapping results) and 3,347 transcripts (sequences with BLAST results only) had no GOs assigned. Of the 38,969 sequences with GO terms assigned, 31,800 (annotated sequences) retrieved reliable functions, leaving 7,169 transcripts with mapping results only.

**Figure 3:**
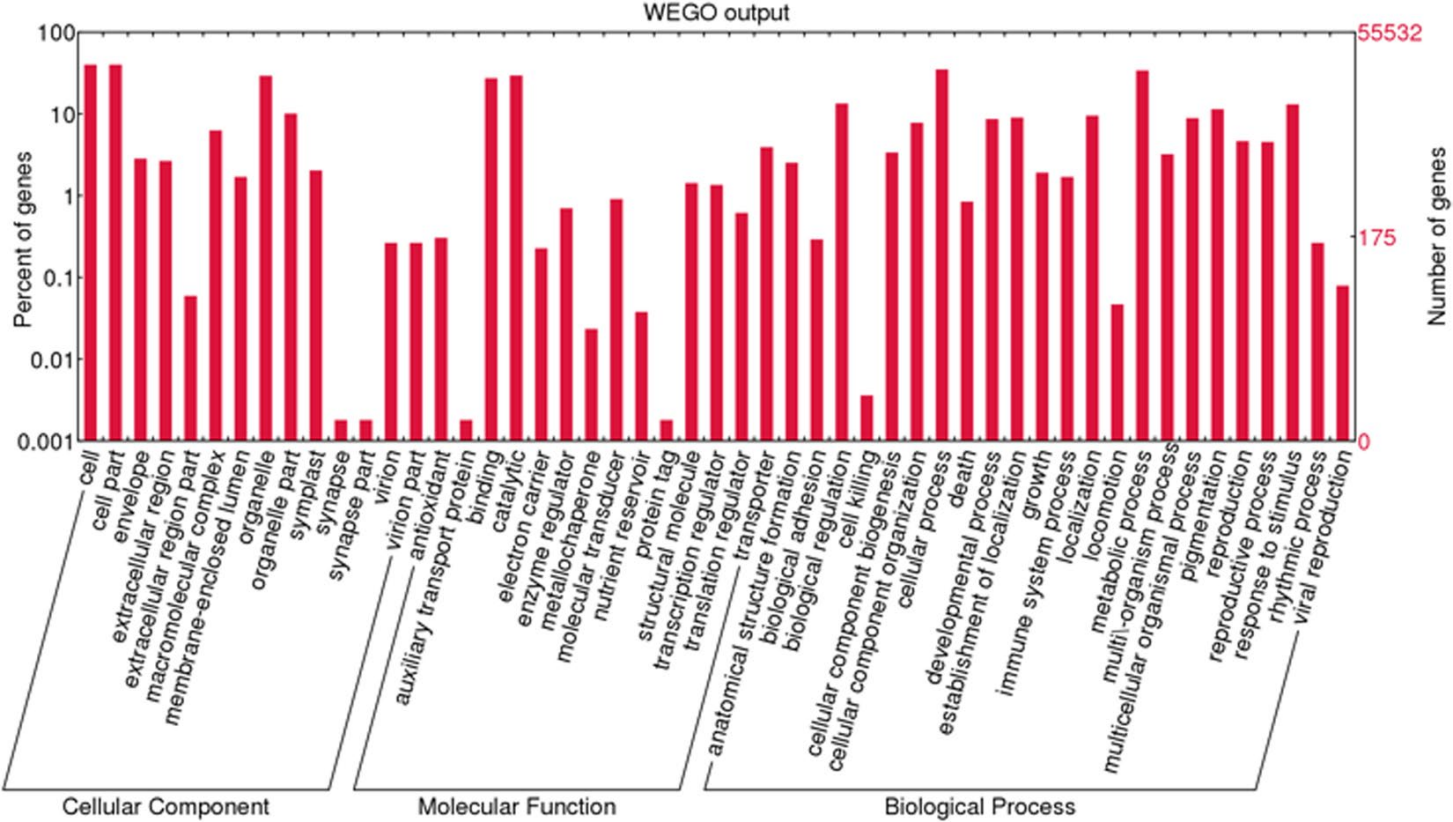
GO classification performed in the transcriptome sequencing dataset.

From the 42,316 transcripts showing positive BLAST hits in the nr database (Fig. 2), 31,800 (75.1%) were annotated with 177,961 GO terms under three main categories: 85,850 corresponded to biological processes, 49,995 to molecular functions and 42,116 to cellular components (Fig. 3). Under the biological process category, cellular processes (19,124 transcripts) were highly represented, followed by metabolic processes (18,422 transcripts) and single-organism processes (16,136 transcripts). Under molecular function, catalytic activity (16,453 transcripts) and substrate binding (15,306 transcripts) were the most highly represented subcategories. Under the cellular component category, cell part (20,737 transcripts), cell (20,737 transcripts) and organelle (16,252 transcripts) were the most highly represented subcategories. Interestingly, under the biological process category, 8,094 transcripts were assigned to GO term “response to stimulus”, in which “response to stress” (4,796 transcripts), “response to chemical” (4,167 transcripts), and “response to abiotic stimulus” (3,362 transcripts) accounted for the most highly assigned GO terms. Distribution of the GO terms under the three categories on level two is shown in Figure S3.

#### KEGG classification

**Figure 4:**
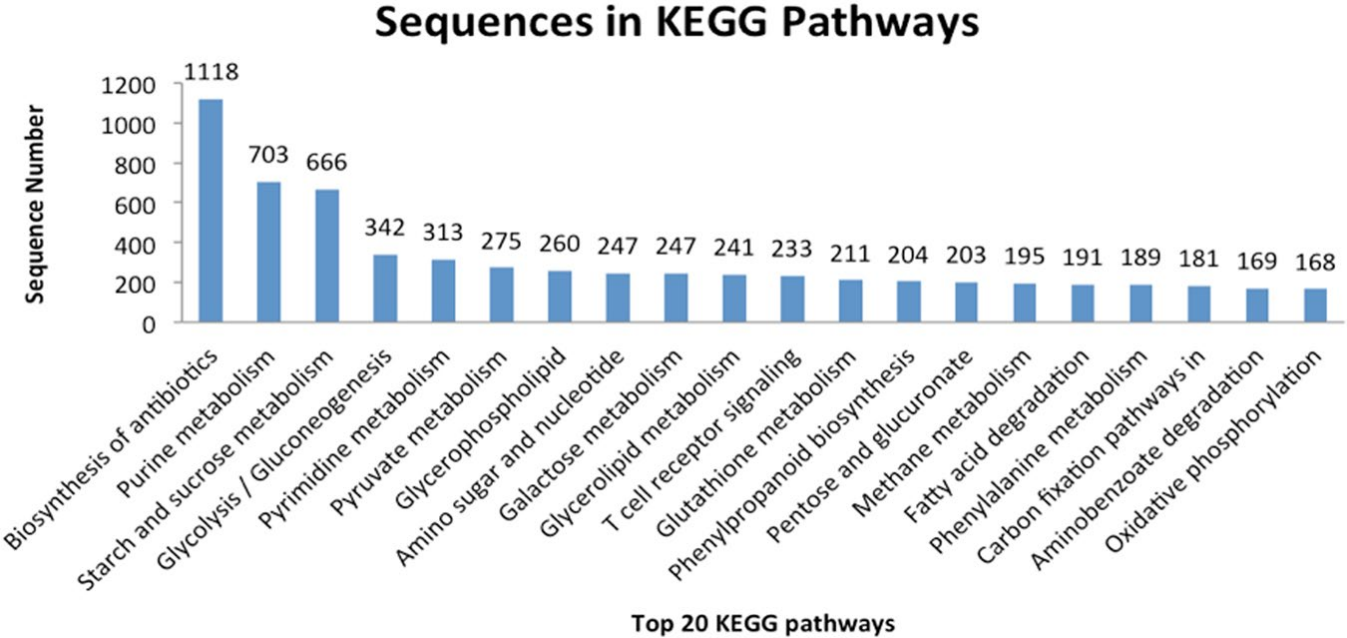
Top 20 KEGG pathways assigned for the assembled transcripts. Top 20 KEGG pathways are listed on x-axis in rank order according to the number of sequences (y-axis) assigned.

31,800 transcripts were annotated with protein functions, and 15,772 were assigned to 143 KEGG pathways using Blast2GO (Table S1). Of the top 20 KEGG pathways with the most assigned transcripts, biosynthesis of antibiotics (1,118 transcripts) was the most represented category. The second most highly represented pathway was purine metabolism (703 transcripts), followed by starch and sucrose metabolism (666 transcripts), glycolysis/gluconeogenesis (342 transcripts) and pyrimidine metabolism (313 transcripts) (Fig. 4). Of the 31,800 annotated transcripts, 102 transcripts encoding 24 enzymes were involved in the terpenoid backbone biosynthesis and metabolism pathways, among which 36 transcripts were involved in the mevalonate pathway (MVA) and 26 were involved in the MEP pathway (Table S2). Geranyl diphosphate (GPP) synthase was the most highly represented enzyme, related to 23 transcripts, followed by HMG CoA reductase (HMGR), farnesyl diphosphate (FPP) synthase and geranylgeranyl diphosphate (GGPP) synthase, which were represented by 17, 16 and 12 transcripts, respectively. All the enzymes involved in the MVA and MEP pathways were identified (Figure S4). In addition, 48 transcripts encoding 15 enzymes were involved in the steroid biosynthesis pathway. The first two enzymes (squalene synthase and squalene monooxygenase) in the steroid biosynthesis were represented by 3 and 6 transcripts, respectively. However, when searched against the KEGG pathway database in Blast2GO, the online TKR ESTs only identified 56 sequences encoding 10 enzymes in the terpenoid backbone biosynthesis pathway, among which 45 sequences were assigned to all 6 enzymes of the MVA pathway. No enzymes of the MEP pathway were represented in the TKR ESTs (Figure S5).

### TK homologues of rubber yield-related genes

Fifty enzymatic and structural proteins are known to be putatively or actually related to rubber yield. Our TK root transcriptome sequences generated 882 significant BLAST hits under e-value 1e-5 for 49 of these proteins with BLASTx. Chicory 1-SST generated no significant TK homologues even though the more closely related *Taraxacum officinale* 1-SST did. A total of 472 transcripts representing 47 proteins with greater than 50% identity were filtered by decreasing the e-value to 1e^−10^. All the gene homologues of the CPT, SRPP and REF families as well as the terpenoid biosynthesis related enzymes were identified (Table S3). CPTs (CPT1-3), SRPP and REF families were represented by 2 homologues (9 transcripts), 4 homologues (7 transcripts) and 4 homologues (6 transcripts), respectively. In the terpenoid biosynthesis pathway, HMGR was the most highly represented gene with 9 homologues (18 transcripts). These results were similar with the ones identified by KEGG pathway analysis. In the inulin biosynthesis pathway, 1-FFT, 1-FEH (I & II) and 1-SST were represented by 9 homologues (37 transcripts), 5 homologues (37 transcripts) and 5 homologues (16 transcripts), respectively.

### Differential Expression Analysis

**Figure 5:**
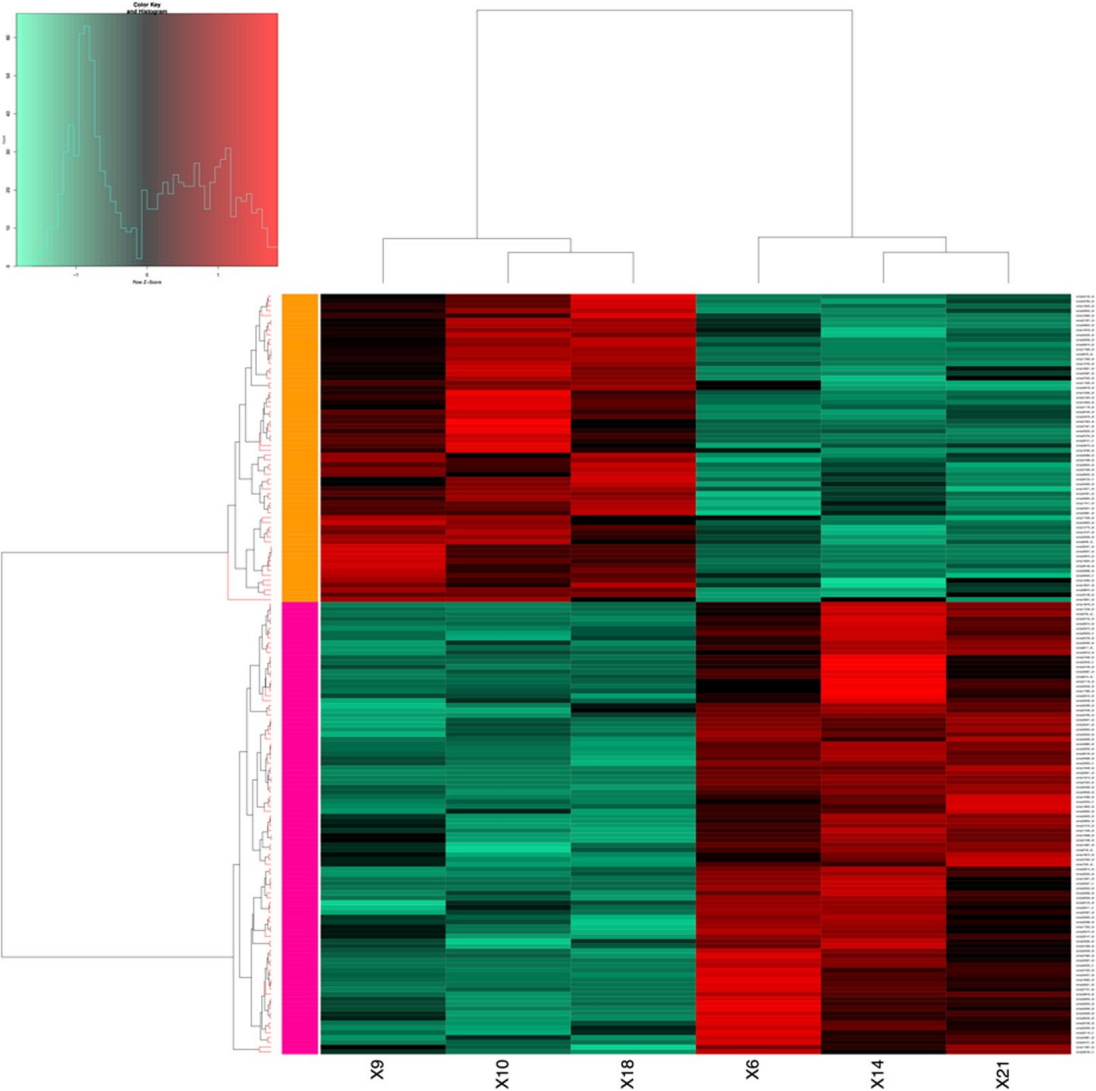
Differentially expressed genes between high rubber and low rubber groups. Red- and green-colored ones were upregulated and downregulated genes, respectively.

The gene expression patterns for each sample were calculated as transcripts per million (TPM). A total of 21,802 genes with detectable expression levels were obtained from two groups of plants with high and low root rubber concentration (Table S4). A comparison between the high and low rubber groups resulted in 792 significantly (p-value < 0.05) differentially expressed (DE) transcripts after false discovery rate (FDR) correction and adjustment for individual variation (Table S5). After removal of genes only counted in one sample, a total of 338 transcripts with posterior fold change (PosteriorFC) values ≥2 or ≤0.5 were retained while all others were filtered out. The hierarchical clustering of the DE transcripts using 158 transcripts with consistent expression patterns showed that 94 (59.5%) of these transcripts were upregulated in high rubber samples (with a PosteriorFC higher than 9), and 64 (40.5%) were upregulated in low rubber samples (with a PosteriorFC of 0.34, Fig. 5). Functional annotation for all DE transcripts was attempted in Blast2GO (Table S6), but 74 (46.8%) generated no annotation results, including the five most highly differentially expressed ones.

### qRT-PCR

**Figure 6:**
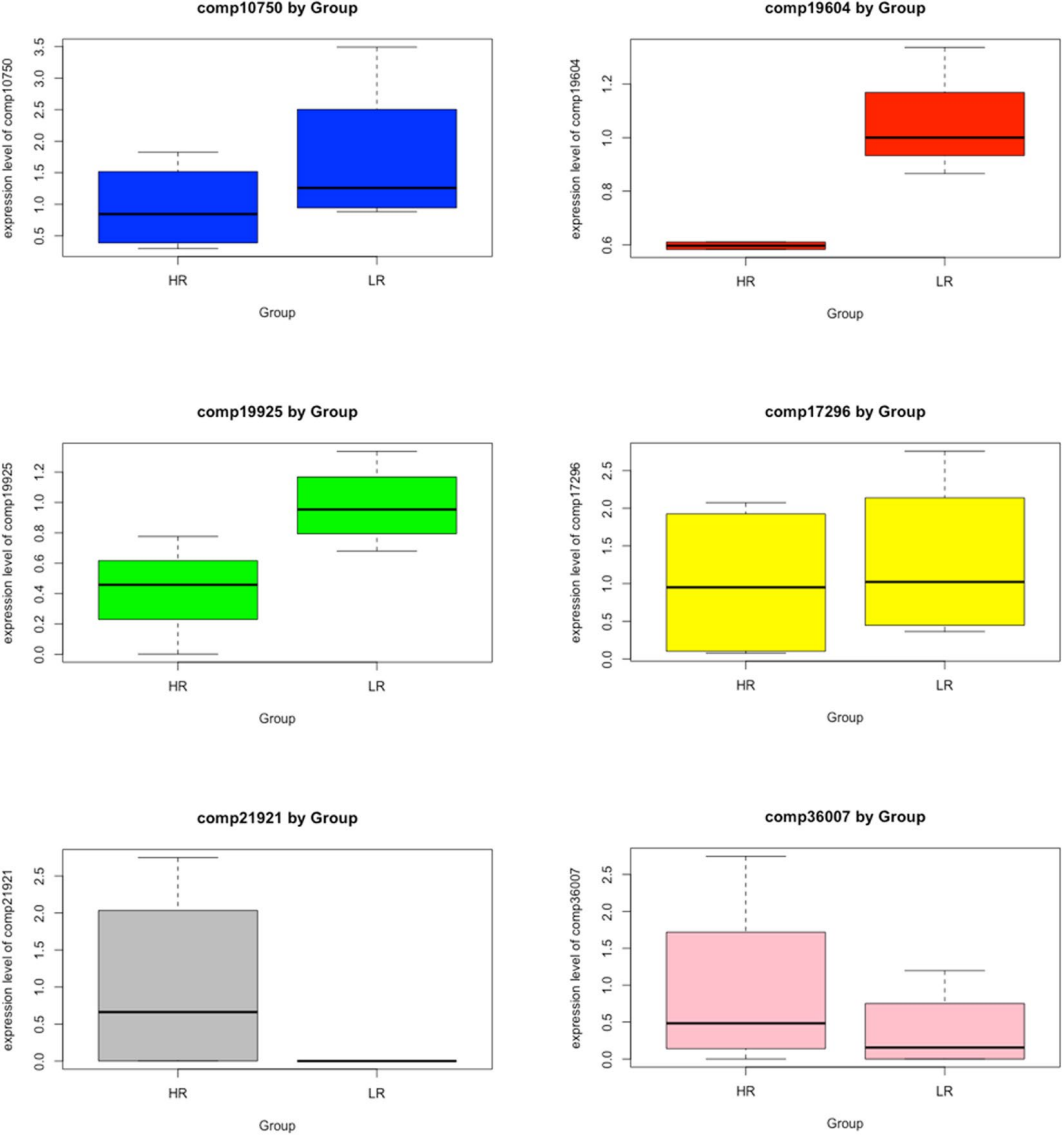
Confirmation of differentially expressed genes using qRT-PCR. Out of six differentially expressed genes we reverse-transcribed, three of them (comp10750, comp19604, comp19925) were upregulated in LR samples, one (comp17296) was slightly upregulated but not obvious, another two (comp21921 and comp36007) contradicted the transcriptome results.

Six out of 10 highest differentially expressed genes were selected for qRT-PCR. *ACTB* (β-actin) *and EF1A* (elongation factor 1α) were used as reference housekeeping genes^22^. All six genes were expected to be upregulated in LR samples based on the transcriptome data. However, qRT-PCR only confirmed four of them (Fig. 6) even though ANOVA p-value failed to prove significant differences between groups. Comp21921 was only expressed in sample 1004 within the HR group and comp36007 was highly expressed in HR samples, which contradicted the transcriptome result. Comp10750, comp19925 and comp19604 were upregulated while comp17296 was only slightly upregulated in LR samples. Half of the developed DE transcripts were confirmed by qRT-PCR in terms of the expression levels obtained from transcriptome data.

### SNP Detection

**Figure 7:**
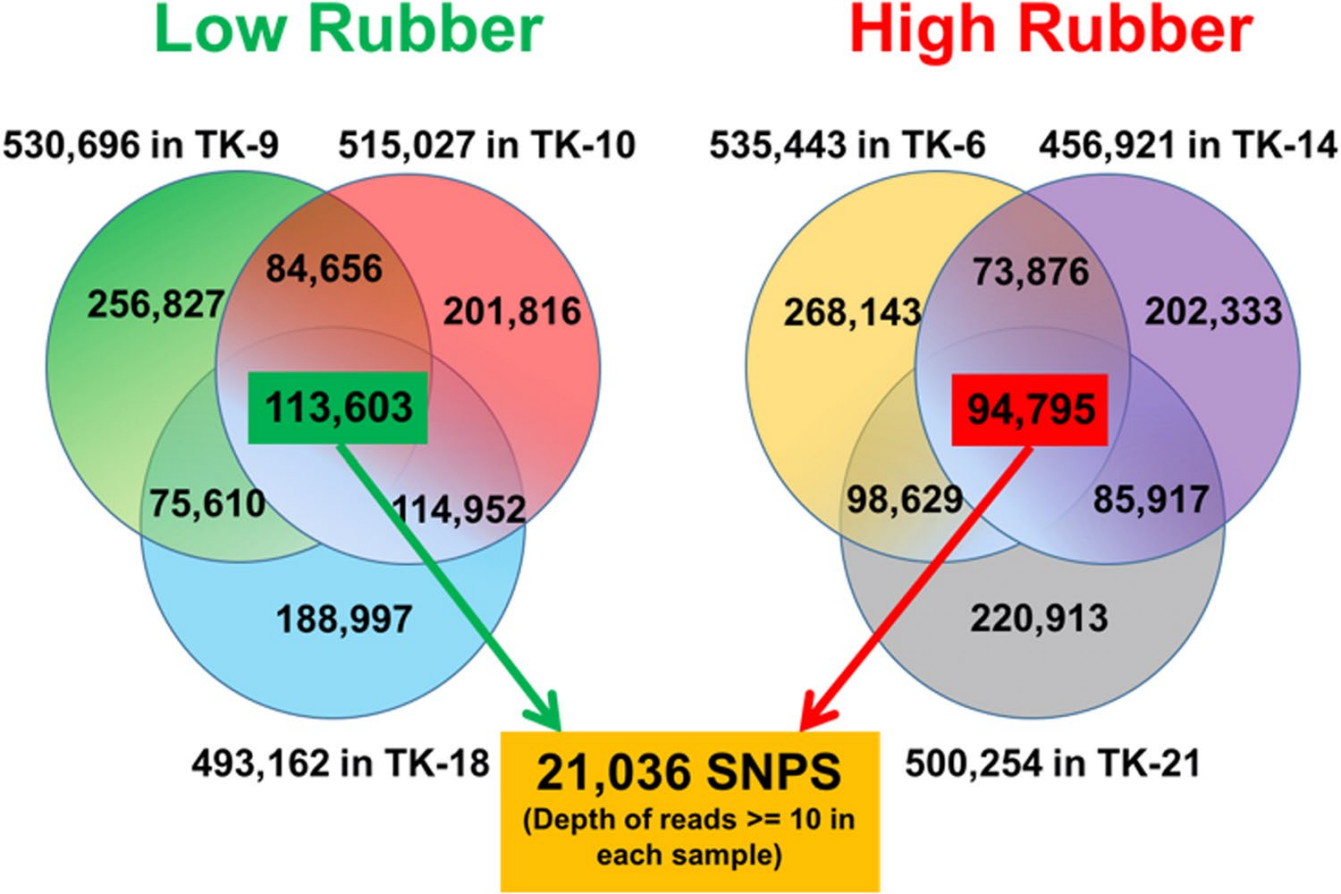
21,036 putative SNPs identified in high rubber and low rubber plants. A total of 113,603 and 94,795 SNPs were first found within the LR and HR group, respectively. Then homozygous SNPs (AA or aa) in all the three samples within either one or both groups were identified and these formed 16,891 SNPs. A total of 4,145 heterozygous SNPs (Aa) in both groups were also identified. Thus, a total of 21,036 SNPs including homozygous and heterozygous types were obtained from HR and LR plants.

A total of 113,603 SNPs were found in the low rubber accessions and 94,795 SNPs were initially identified in the high rubber accession and then homozygous SNPs (AA or aa) in all the three samples within either one or both groups were identified and these formed 16,891 SNPs. A total of 4,145 heterozygous SNPs (Aa) in both groups were also identified. Thus, a total of 21,036 putative SNPs including homozygous and heterozygous types were obtained from HR and LR plants with read depth greater than 10 (Fig. 7). Of these putative SNPs, 16,891 were homozygous in either the high rubber or low rubber group, but 4,145 were heterozygous in both HR and LR groups (Table S7). Out of 21,036 SNPs, 13,107 (62.3%) were transition SNPs while 7,929 (37.7%) were transversion SNPs (Table S8). Among the transversion SNPs, A ↔ T was the most common with 2,378 SNPs, while C ↔ G was the least common with 1,794 SNPs.

#### SNPs on rubber-related homologues and differentially expressed transcripts

Among the 472 transcripts showing significant BLAST hits when searched against the 50 publically available rubber-related proteins, 94 of them carried 112 SNPs. After removing replicated SNPs appearing in the same protein families, fifty SNPs were assigned on those 94 rubber-related transcripts (Table S9) but none of these transcripts showed differential expression. Among the 158 differentially expressed transcripts, 36 of them carried 117 SNPs (Table S10). No SNPs were found in the five most highly differentially expressed transcripts, which were comp17296_c0, comp19604_c0, comp34875_c0, comp21921_c0 and comp28151_c1. None of these transcripts generated a protein function description after annotation.

## Discussion

A comparison of assembly results between non-normalized and normalized data found that the normalized data performed better both in N50 and OHR, indicating that these data could generate assemblies with better quality and completeness than non-normalized data (Figure S1). The quality of the normalized data is supported by a N50 value of 1827bp, which is higher than the non-normalized values of 720 bp and 1545 bp measured in *Hevea* ^23^ and *Cichorium intybus* (chicory)^24^, respectively. Thus, normalization can improve data computing efficiency without impairing data quality and completeness. While normalization may mitigate representation bias in the assembly RNA-Seq data, due to highly variable degrees of coverage, it may also introduce artifacts; however, these can be minimal^25^.

When comparisons were made between our normalized transcriptome dataset and the online non-normalized TKR EST database, 80.9% of the unigenes in the TKR-EST database were covered by our transcriptome dataset. However, 93.2% of the unigenes in the transcriptome were not covered by the TKR-ESTs. Also, transcript length is usually used as a significant predictor of the presence or the absence of a significant BLAST hit in the NCBI databases^26^. The mean sequence length of transcripts with significant BLAST hits in our study was 1704bp, which was greater than similar transcriptome analyses in *Hevea* (536 bp)^23^, stevia (969 bp)^20^ and safflower (679 bp)^21^. Overall, these data suggest that our assembly is a major contribution to the Asteraceae and rubber producing plant species. The transcriptome identified a much larger number of predicted genes than existing TKR-ESTs; furthermore, the greater N50 statistic of 1977bp than 744 bp of the TKR-ESTs, demonstrate that the transcriptome provides  an improved genomic resource for the under-represented TK species and *Taraxacum* genus.

When we analyzed our transcriptome using top-hit species analysis, we did not identify obvious close relatives in the Asteraceae, with *Lactuca sativa* (lettuce) being the sole exception at ranking position 19 (of 30). Similar outcomes also were found in other Asteraceae family species^20, 21^ and other rubber-producing plants^23^. We believe this is an artifact of the top-hit species analysis being heavily skewed away from identifying species with limited publically available genomic resources. Several species with an overrepresentation of genomic data, such as *Vitis vinifera, Glycine max*, and *Solanum tuberosum*, resulted in a much higher chance of BLAST hit alignment with the TK transcriptome than near relatives even though they are not closely related taxonomically. Thus, this type of analysis provides limited information for species in early development of genomic resources; although it will improve as additional genomic resources for other species are being developed.

Plant secondary metabolites are the direct indicators of biochemical activities and provide the readout of cellular state^27^. Therefore, we subjected out transcriptome to KEGG pathway analysis. This analysis showed that purine metabolism, starch and sucrose metabolism, glycolysis/gluconeogenesis metabolism and pyrimidine metabolism were highly represented in TK plants at the onset of winter. Similar results also were found in *Hevea* under cold-stressed and suboptimal growing conditions^28^. In addition, allantoin or allantoic acid is formed through oxidative purine decomposition^29^ and they play an important role in the storage and translocation of nitrogen in leguminous plants^30^. Therefore, purine metabolism may be related to nitrogen assimilation in TK. Moreover, both precursors of the MVA pathway (Acetyl-CoA) and the MEP pathway (Glyceraldehyde-3-phosphate) were produced from the glycolysis/gluconeogenesis pathway^23^, which justifies the representation of glycolysis/gluconeogenesis metabolism. Additionally, all the enzymes in the MVA and MEP pathways of the terpenoid backbone biosynthesis were identified by transcriptome annotation. Compared to the fact that none of the enzymes in MEP pathway were identified in TKR ESTs, the TK transcriptome again reflected the expansion of TK current genomic resources.

In addition to the enzymes involved in terpenoid backbone biosynthesis, homologues of CPTs, CPTL, SRPP and REF were also identified in the TK transcriptome with identity percentages of 100%, 98.00%, 51.22%, and 51.08%, respectively. The homologues to the corresponding protein families of *Taraxacum brevicorniculatum* (TB) generated the highest identity percentage (e.g. homologues to TbCPT1, LsCPT1 and HbCPT1 with identity percentages of 97.08%, 91.02% and 58.67%, respectively), followed by lettuce and *Hevea*, indicating the relative relationship between TK and these species, which corresponds to the phylogenetic relationship reported by Zhang *et al*.^11^. SRPP and REF share common sequences, consistent with a previous phylogenetic analysis that REF and SRPP are homologous proteins originating from a common ancestor gene, and that they belong to a larger plant family of stress-related proteins^31^.

In TB plants (a close relative of TK, which produces less rubber than TK^11^) grown for three months in growth chambers at 18 °C, rubber and inulin levels were negatively correlated, suggesting a relationship between these two assimilate sinks^17^. Inulin biosynthesis involves two enzymes: sucrose:sucrose 1-fructosyltransferase (1-SST) (EC 2.4.1.99)^32^ and fructan:fructan 1-fructosyl transferase (1-FFT) (EC 2.4.1.100)^33^. 1-SST initiates inulin polymerization, whereas polymerization is catalyzed by 1-FFT which transfers fructose moieties from larger fructans to make inulin polyfructans much larger and complex^33^. 1-fructan exohydrolase (1-FEH) breaks down inulin into sucrose and fructose and then acid invertase breaks down sucrose to glucose and fructose^34^. Chicory, a crop cultivated for inulin, has two different 1-FEH forms (1-FEH I and II), and 1-FEH II can be further differentiated into two isoforms (1-FEH IIa and IIb)^33^. Inulin degradation in chicory is induced at the end of the growing season at the onset of winter when 1-SST has almost disappeared, while 1-FFT activity is still high and 1-FEH is induced^33^. Our TK transcriptome contains homologues to all the chicory inulin enzymes described above, except for the 1-SST, indicating that inulin biosynthesis and degradation occur in a very similar pattern to chicory.

No differentially expressed genes were found to be directly related to the rubber particle proteins, such as REF and SRPP, in TK, although these are more highly expressed in rubber producing latificers in *Hevea* than in other tissues^35^ and REF was more highly expressed in high yielding rubber clones than in low yielding ones^36^. However, this may simply result from the relatively low abundance of rubber particles. The CPT gene family also was not differentially expressed in these studies^35, 36^ nor in TK (this study). Similar results were found in guayule^37^. The lack of differential expression of these putative rubber related genes casts doubt on a strong direct role in rubber biosynthesis. Also, although REF and SRRP expression were related to higher latex yield^36^ in *Hevea*, gene expression levels of these genes and of those for the CPT family did not correlate with rubber transferase activity^35–37^, either in *Hevea* or guayule. The absence of function annotation in the five transcripts (query length ranging from 390 to 1361 bp) with highest difference in expression level demonstrated that novel genes may exist. Only comp17296_c0 generated BLAST hit (not significant) to only 20-amino acid in length sequence of carboxyl-terminal protease, which is largely hydrophilic^38^. We also observed that the expression level of 1-FFT (which did not pass filtering for significant differential expression of 0.5 ≤ PosteriorFC ≤ 2, between the high and low groups) appeared to be upregulated in the low rubber group (Table S11).

Among the six differentially expressed genes we selected to design primers for qRT-PCR, three of them (comp17296_c0, comp19604_c0, comp21921_c0) generated no protein functions and qRT-PCR only confirmed the differential expression in comp19604_c0 while comp21921 was only expressed in a single HR sample (TK1004) and comp17296 was uniformly expressed between the two groups. This might be due to multiple reasons: 1) sequencing or assembly error may occur; 2) the samples used in qRT-PCR are not the same as those that were RNA-Sequenced. 3) harvest time and growing conditions are not the same for qRT-PCR samples and RNA-Seq samples, leading to the variation in gene expression; 4) sample size and replications in either RNA-Seq or in qRT-PCR may be insufficient; 5) technical noise may occur during PCR reactions. Moreover, it is known that rubber accumulation is highly influenced by environmental effects and that strong genotype × environment interactions occur in TK^39^, guayule^40^ and *Hevea* ^41^; such effects may have overwhelmed rubber related gene expression differences contributing to TK rubber yield variation. This may be because important genes involved in TK rubber biosynthesis are expressed at very low levels or are yet unidentified (such as those encoding the rubber transferase complex itself).

Other than the three transcripts without gene annotation, comp10750_c0, comp19925_c0 and comp36007 were annotated as GRAS family transcription factor, disease resistance protein rpm1-like and rho guanyl-nucleotide exchange factor, respectively. All of them, except comp36007, confirmed the upregulated expression in LR samples. GRAS proteins, named after the first three members: GIBBERELLIC-ACID INSENSITIVE (GAI), REPRESSOR of GAI (RGA) and SCARECROW (SCR)^42^, are a family of plant-specific proteins reducing arbuscular mychrrhization during arbuscule development^43^. Rpm-1 like protein is a member of nucleotide binding site and carboxyl-terminal leucine-rich repeats (NBS-LRR) class in plant disease resistance (R) gene products from *Arabidopsis thaliana*, which confers resistance to *Pseudomonas syringae* expressing avirulence (avr) gene products including *avrRpm1* or *avrB* ^44^. The expression of these genes may suggest that plants were triggering some defense mechanisms in response to pathogen attack. Combined with the fact that phytoalexins are a group of isoprenoids conferring antimicrobial function^45^, the higher expression of disease resistance genes in LR samples may suggest a negative relationship between rubber biosynthesis and disease defense when competing for the same precursors (e.g. mevalonate) synthesized from MVA pathway. Likewise, the analysis of draft genome sequence of *Hevea* also identified over 800 genes related to disease resistance, but they didn’t delve into the relationship of rubber production and the expression of these genes^46^.

An interesting gene upregulated in the low rubber group was squalene monooxygenase (or squalene epoxidase), which catalyzes the first oxygenation step in sterol biosynthesis and is one of the rate-limiting enzymes in sterol biosynthesis^47^. When rubber production was inhibited in 3-month-old TB plants, HMGR was also inhibited and fewer rubber particles were made. In response to this imposed limitation on IPP consumption, sterol synthesis was induced^34^. The upregulated squalene monooxygenase in the low rubber TK group may reflect the effects in the low rubber phenotype. Either way, these data suggest that improved rubber biosynthesis in TK may be achieved by overexpression of HMGR coupled with downregulation of squalene monooxygenase, restricting the flow of assimilate to sterols.

The diploid genome size of TK is 2.4 GB^9^, and the total SNPs detected translate to 1 SNP per 153 bp. This frequency is similar to the SNP frequencies that were previously reported for *Hevea* (1 SNP per 125 bp)^23^ and *Helianthus annuus* (sunflower) (1 SNP per 140 bp)^48^ but is distinct from safflower (1 per 303 bp)^21^ or chicory (1 SNP per 1068 bp)^24^. The usual frequency of SNPs reported for plant transcriptomes is about 1 SNP every 100–300 bp^49^. SNP frequency is positively correlates with diversity and TK is clear more genetically diverse than safflower or chicory. A similar conclusion also can be drawn from the observed heterozygosity based on SNPs. Of the observed 21,036 SNPs, only 1990 (9.46%) showed homozygous divergence between high and low rubber groups. More than 90% of SNPs showed heterozygosity at least in one group. Since diversity level is proportional to heterozygosity, we can further conclude that TK was considerably more diverse than safflower^50^, which contains 20-30% less nucleotide diversity compared to its wild species *Carthamus palaestinus*, and chicory^24^ with only 68% heterozygous SNPs. In addition, the transition SNPs were generally observed at a higher frequency as expected, suggesting that transition mutations are better tolerated than transversion mutations during natural selection of TK. This may be due to synonymous mutations in protein-coding sequences, as has been seen in other species such as *Hevea* ^23^ and chicory^24^.

Because transcripts/homologues corresponding to genes that are involved in rubber substrate production were identified using KEGG annotations and homologue BLASTx queries, we also searched for SNPs in these sequences. Three genes encoding hydroxymethylglutaryl-CoA synthase (HMGS), mevalonate kinase (MVK) and diphosphomevalonate decarboxylase (MVD) in the MVA pathway and one gene encoding IDP isomerase in the downstream terpenoid pathway did not contain any putative SNPs. *Hevea* does have SNPs in these genes^23^. This difference may be because the homologous genes conserved in one species are less conserved in another species even if they perform similar functions^51^. In TK, more SNPs were found in the genes involved in inulin biosynthesis than in rubber production, indicating that rubber-related genes are more conserved. The limited number of plants considered in this study does not allow strong conclusions to be drawn about the actual allelic variation of rubber-related genes. However, the identified markers provide a foundation from which to explore polymorphisms in breeding germplasm of TK, as well to validate and retrain markers useful for marker assisted selection.

## Conclusion

We have used RNA sequencing to generate a comprehensive transcriptome database for TK roots, considerably expanding on the currently available TKR EST database as well as providing a broad characterization of expressed genes in TK root tissue. The identification of diverse genes showing functional significance through BLAST searches, GO analysis, KEGG pathway analysis, and WEGO gene function distribution analysis indicates that a robust transcriptome sequence assembly was completed. The high homology between TK homologues of genes possibly related to rubber yield in other species suggests a conservative evolution of the genes controlling rubber biosynthesis. Genes were identified which were differentially expressed in high rubber and low rubber accessions. The identification of a large set of genetic variants provides a foundation for future genetic analysis and applied breeding efforts. Our results indicate that the characterization of transcripts within a non-model species can be effectively realized by assembling short reads generated through RNA Illumina sequencing, confirming several other recent studies. In summary, an extensive genomic resource for TK has been developed, which adds useful information to the limited genetic data developed for TK, and will aid in subsequent efforts on QTL or association mapping studies, marker-assisted selection, as well as for functional genomics via gene editing strategies and comparative genomics among/within *Taraxacum* species.

## Materials and Methods

In order to generate a complete transcriptome profiling of TK roots, samples from an OSU bulked seed lot of USDA TK germplasm collections^52^ were phenotyped for rubber content (details below), and six samples were RNA sequenced in December 2013. *De novo* assembly, transcript functional annotation and biological interpretation were performed. TK homologue analysis and differentially expressed genes putatively related to rubber biosynthesis were also identified. Single Nucleotide Polymorphisms on these genes were discovered for further use in molecular breeding of TK.

### Plant material and RNA extractions

Twenty TK plants grown in the Muck Crops Agricultural Research Station in Ohio were harvested in December of 2013 when the rubber induction was expected to be high^53^. All the harvested root tissue was immediately frozen in liquid nitrogen and stored at −80 °C until RNA extraction. After rubber quantification with three technical replications by Accelerated Solvent Extraction (ASE)^39^, 3 relatively high rubber plants (TK6, TK14, and TK21 with an average rubber concentration of 44.35 ± 4.48 mg/g) and 3 low rubber plants (TK9, TK10, and TK18 with an average rubber concentration of 15.03 ± 3.09 mg/g) were selected for RNA-Seq (Table S11). Total RNA was extracted from these 6 samples using an RNeasy Mini Kit (Qiagen, Germany) following the manufacturer’s instructions. The concentration of total RNA was determined using a NanoDrop ND-1000 spectrophotometer (Thermo Scientific, USA). High-quality RNA was provided for RNA-Seq library construction and Illumina sequencing (Table S12).

### cDNA library construction and Illumina sequencing

cDNA libraries were constructed from those six RNA samples using the Illumina TruSeq RNA Preparation Kit according to manufacturer’s instructions (Illumina, San Diego, CA). Briefly, the basic processing steps were a magnetic bead based isolation of polyA mRNA, chemical fragmentation, double stranded cDNA synthesis, end polishing and adapter ligation, followed by PCR enrichment of the library. Each of six samples were barcoded and pooled according to the experimental design and a paired-end 2 × 100 base sequencing run was performed on the Illumina HiSeq. 2000 sequencer (Illumina, San Diego, CA) at Beijing Genomics Institute (BGI).

### Data filtering and *de novo* assembly

RNA-Seq reads obtained from Illumina HiSeq. 2000 were quality evaluated using the FASTX-Toolkit (Hannon Lab; http://hannonlab.cshl.edu/fastx_toolkit/), the quality cut-off score used was 30 (-q). Reads were deposited in the National Center for Biotechnology Information Sequence Read Archive under accession number: TK6 (SRR5181667); TK9 (SRR5181665); TK10 (SRR5181664); TK14 (SRR5181663); TK18 (SRR5181662); and TK21 (SRR5181661). Filtered reads then went through Trinity^25^ (version 0.0.2), both before and after normalization. N50 and ortholog hit ratios (OHR) were used to compare and evaluate assembly quality and completeness between the normalized data subset and the whole dataset. Paired reads passing the filter were concatenated using Concatenate datasets (version 1.0.0) in both the right and left direction. Paired reads were processed by averaging statistics between pairs and retaining linking information. Reads statistics were generated in parallel for paired reads. The normalized data subset was then assembled into transcripts using *de novo* assembly tool Trinity. Transcript abundancies were then calculated using RSEM version 1.1.17^54^ with default settings using the pool of non-normalized reads. Transcripts with a Transcript Per Million (TPM) of less than one were removed in all samples using Filter and seq_filter_by_id (version 1.1.0).

### Characterization via similarity searches and annotations

The assembled transcripts and online ESTs also were searched against the NCBI non-redundant (nr) protein database using BLASTx on Ohio Supercomputer Center^55^ with a cut-off e-value of 1e^−5^. The Blast2GO 3.0^56^ program then was used to obtain gene ontology (GO) and Kyoto Encyclopedia of Genes and Genomes (KEGG) annotations. The online-based software WEGO was employed to compare and contrast the GO classifications of the annotated transcripts between transcriptome sequencing data and the online TK root (TKR) EST database. The transcripts representing enzymes involved in the rubber biosynthesis pathway were identified. In addition, a local TKR database was constructed using 16,441 TKR ESTs downloaded from GenBank (accession numbers DR398435 to DR403165 and GO660574 to GO672283). In order to perform a BLASTn search with a cut-off e-value of 1e^−20^ for the assessment of our transcriptomic contributions to the TKR ESTs, unigenes of each dataset were assembled into transcripts using CAP3^57^ with the default settings prior to SNP identification. Moreover, a local database of 50 rubber-related proteins was established and a BLASTx search with a cut-off e-value of 1e^−5^ for the homolog identification in our transcripts was performed. The homologues were then filtered by increasing e-value to 1e^−10^ and setting the identity percentage threshold to 50.

### Differential gene expression analysis

After the filtering and counting of mapped reads by RSEM v1.1.17, differential expression values were computed with EBseq (version 1.3.3)^58^ by using a Bayesian approach to estimate isoform expression. Comparisons were performed to find the particular genes which distinguish the high rubber and low rubber accessions. Count data were normalized by estimating a scaling factor for each contig in EBseq. Posterior Fold Change (FC) of each differentially expressed gene was obtained using an empirical Bayes hierarchical model. Comparisons were accepted as significant at an FDR adjusted value of 0.05. For visualization of the significant comparisons, heatmaps of the significant differentially expressed genes were produced with the heatmap.2 function from gplots CRAN library in R (v3.3.2, 2016)^59^. Hierarchical clustering of individual samples with 10,000 bootstrap replications was performed with the R package pvclust^59^ and heatmaps were sorted accordingly. The above procedures were repeated after the manual removal of dubious transcripts, which showed inconsistent patterns of expression within plants of a given group (HR vs LR). Thus, transcripts that were not consistently expressed among the three plants of a given group (i.e. the expression of a given transcript is not statistically similar –no change, upregulated or downregulated– within the three plants in the group, HR or LR) were discarded. Only consistent expression levels across plants within groups were considered for clustering.

### qRT-PCR

Four TK clones (two with relatively high rubber, ﻿42.61±4.7mg/g﻿, and two with low rubber, 20.66±2.8mg/g,) from a USDA TK accession germplasm pool^52^ were harvested and transplanted into soil under 4°C for five-days. These four TK clones were unrelated to the six RNA-Seq samples. Approximately 100 mg of TK root tissues were used for total RNA extraction using the RNeasy Plant RNA Kit (Qiagen, Germany) according to the manufacturer’s recommendations and then were treated by DNase I using TURBO DNA-free^TM^ Kit to remove DNA (InvitrogenTM, Carlsbad, CA, USA). First strand synthesis of mRNAs was carried out using ProtoScript II reverse transcriptase and oligo-dT following the manufacturer protocol (NEB). After the synthesis of first-strand cDNA had finished and subsequently diluted five-fold, PCR was performed to analyze the expression pattern of six differentially expressed genes (Table S14), and gene *ACTB* (β-actin) and *EF1A* (elongation factor 1α) were used as reference housekeeping genes^22^. All the nuclear sequences of the designed gene-specific primers were designed using Primer 3.0 software (http://primer3.ut.ee) as seen in Table S14. The qPCR analyses were performed in CFX96 Touch Real-Time PCR Detection System (Bio-Rad, Hercules, CA, USA) using SsoAdvanced^TM^ Universal SYBR Green Supermix in a final volume of 20 μl. The reaction mixture consisted of 10 μl SsoAdvanced universal SYBR® Green Supermix (2x), 2 μl primer (5 μM forward and reverse primers), 2 μl of diluted cDNA, and 6 μl nuclease-free water. The same batch of diluted cDNA (5 ml, corresponding to 50 ng of reverse transcribed RNA) was subjected to qPCR to amplify all candidate genes for mRNA normalization as well as target gene. Five ml of respective cDNAs were used for qPCR analysis of each microRNA. The PCR reaction of each plate must include internal control gene *EF1A* to eliminate variation between plates. Non-template controls (NTC) were also done for each primer pair. All the Real-time PCRs were performed under the following conditions: 5 min at 95^◦^C, and 40 cycles of 15 s at 95 °C and 30 s at 60 °C in 96-well reaction plates (Bio-Rad). The specificity of amplicons was verified by melting curve (disassociation) analysis (60–95 °C) after 40 cycles. All reactions were performed in triplicate. The Bio-Rad CFX96 Manager software (Bio-Rad laboratories, Inc.) was used to perform gene expression analysis.

### SNP detection

The RNA-Seq reads were aligned to previously assembled transcripts using Bowtie2^61^ v2.1.0 with default settings. Based on the alignments, SNP, MNP (multi-nucleotide polymorphism) and insertion/deletion calls were generated using FreeBayes (https://github.com/ekg/freebayes). The following parameters were used to filter SNPs: 1) variant calls with reads depth less than 10 in each sample were discarded. 2) The minimum frequency of the minor allele was 20%; and 3) within each possible nucleotide at the homozygous SNP position, all of its bases at the SNP position are either common in three high rubber accessions or in three low rubber accessions. SNPs on TK homologues and differentially expressed genes were selected.

## Electronic supplementary material

Supplementary Figures

Supplementary Tables

## References

[1] Hayashi, Y. Production of natural rubber from Para rubber tree. *Plant Biotechnol* **26**, 67–70 (2009).

[2] van Beilen JB, Poirier Y (2007). Guayule and Russian dandelion as alternative sources of natural rubber. Crit Rev Biotechnol.

[3] United States. In *The World Factbook* (Central Intelligence Agency, 2016).

[4] Basiron Y (2007). Palm oil production through sustainable plantations. Eur J Lipid Sci Tech.

[5] Cornish K (2017). Alternative natural rubber crops: why should we care?. Technol Innov.

[6] Rivano, F. *et al*. *Hevea brasiliensis* for yield, growth and SALB resistance for high disease environments. *Ind Crop*. *Prod* **44**, 659–670 (2013).

[7] Irogue, V. Effects of White Root Rot Disease on *Hevea brasiliensis (Muell. Arg.)* – Challenges and Control Approach. In N. K. Dhal. & S. C. Sahu. (Eds.), *Plant Science: InTech.* (2012).

[8] McAssey, E. V., Gudger, E. G., Zuellig, M. P. & Burke, J. M. Population genetics of the rubber-producing Russian dandelion (*Taraxacum kok-saghyz*). *PLoS ONE* **11(1)**, (2016). 10.1371/journal.pone.0146417 PMC4703197 26727474

[9] Kirschner J, Štěpánek J, Černý T, De Heer P, van Dijk PJ (2012). Available *ex situ* germplasm of the potential rubber crop *Taraxacum koksaghyz* belongs to a poor rubber producer, *T. brevicorniculatum* (Compositae–Crepidinae). Genet. Resour. Crop Evol.

[10] Arias, M. *et al*. First genetic linkage map *of Taraxacum koksaghyz* Rodin based on AFLP, SSR, COS and EST-SSR markers. *Sci Rep* **6**, 31031 (2016). 10.1038/srep31031 PMC4973268 27488242

[11] Zhang Y, Iaffaldano BJ, Zhuang X, Cardina J, Cornish K (2017). Chloroplast genome resources and molecular markers differentiate rubber dandelion species from weedy relatives. BMC Plant Biol.

[12] Schmidt, T. *et al*. Molecular cloning and characterization of rubber biosynthetic genes from *Taraxacum koksaghyz*. *Plant Mol Biol Rep* **28**, 277–284 (2010).

[13] Qu, Y. *et al*. A lettuce (*Lactuca sativa*) homolog of human Nogo-B receptor interacts with *cis*-prenyltransferase and is necessary for natural rubber biosynthesis. *J Biol Chem* **290**, 1898–1914 (2015). 10.1074/jbc.M114.616920 PMC4303647 25477521

[14] Epping, J. *et al*. A rubber transferase activator is necessary for natural rubber biosynthesis in dandelion. *Nature Plants* **1** (2015).

[15] Cornish K (2001). Biochemistry of natural rubber, a vital raw material, emphasizing biosynthetic rate, molecular weight and compartmentalization, in evolutionarily divergent plant species (1963 to 2000). Nat Prod Rep.

[16] Gronover., C. S., Wahler., D., & Prüfer., D. Natural Rubber Biosynthesis and Physic- Chemical Studies on Plant Derived Latex. In MagdyElnashar (Ed.), *Biotechnology of Biopolymers.* (2011)

[17] Post, J. *et al*. Laticifer-specific *cis*-prenyltransferase silencing affects the rubber, triterpene, and inulin content of *Taraxacum brevicorniculatum*. *Plant Physiol.* **158**, 1406–1417 (2012). 10.1104/pp.111.187880 PMC3291264 22238421

[18] Conesa, A. *et al*. A survey of best practices for RNA-seq data analysis. *Genome Biol* **17**, 13 (2016). 10.1186/s13059-016-0881-8 PMC4728800 26813401

[19] Hansey, C. N. *et al*. Maize (*Zea mays L*.) genome diversity as revealed by RNA-sequencing. *PLoS ONE* **7** (2012). 10.1371/journal.pone.0033071 PMC3306378 22438891

[20] Chen, J. *et al*. RNA-Seq for gene identification and transcript profiling of three *Stevia rebaudiana* genotypes. *BMC Genomics* **15**, 571 (2014). 10.1186/1471-2164-15-571 PMC4108789 25001368

[21] Liu, X. *et al*. *De Novo* sequencing and analysis of the safflower transcriptome to discover putative genes associated with Safflor Yellow in *Carthamus tinctorius L*. *Int J Mol Sci* **16**, 25657–77 (2015). 10.3390/ijms161025657 PMC4632820 26516840

[22] Kozera B, Rapacz M (2013). Reference genes in real-time PCR. J Appl Genet.

[23] Mantello, C. C. *et al*. *De novo* assembly and transcriptome analysis of the rubber tree (*Hevea brasiliensis*) and SNP markers development for rubber biosynthesis pathways. *PLoS ONE* **9**, (2014). 10.1371/journal.pone.0102665 PMC4105465 25048025

[24] Testone, G. *et al*. Insights into the sesquiterpenoid pathway by metabolic profiling and *de novo* transcriptome assembly of stem-chicory (*Cichorium intybus* cultigroup “Catalogna”). *Front Plant Sci*. **7** (2016). 10.3389/fpls.2016.01676 PMC5099503 27877190

[25] Haas, B. J. *et al*. *De novo* transcript sequence reconstruction from RNA-seq using the Trinity platform for reference generation and analysis. *Nat Protoc* **8**, 1494–512 (2013). 10.1038/nprot.2013.084 PMC3875132 23845962

[26] Christmas MJ, Biffin E, Lowe AJ (2015). Transcriptome sequencing, annotation and polymorphism detection in the hop bush, *Dodonaea viscosa*. BMC Genomics.

[27] Li K, Wang X, Pidatala VR, Chang CP, Cao X (2014). Novel quantitative metabolomic approach for the study of stress responses of plant root metabolism. J Proteome Res.

[28] Silva, C. C. *et al*. Leaf-, panel- and latex-expressed sequenced tags from the rubber tree (*Hevea brasiliensis*) under cold-stressed and suboptimal growing conditions: the development of gene-targeted functional markers for stress response. *Mol Breed* **34**, 1035–1053 (2014). 10.1007/s11032-014-0095-2 PMC4162974 25242886

[29] Fujihara, S. & Yamaguchi, M. Effects of allopurinol [4-hydroxypyrazolo(3,4-D) pyrimidine] on metabolism of allantoin in soybean plants. *Plant Physiol* **62**, 134–138 (1978). 10.1104/pp.62.1.134 PMC1092072 16660452

[30] Diaz-Leal JL, Galvez-Valdivieso G, Fernandez J, Pineda M, Alamillo JM (2012). Developmental effects on ureide levels are mediated by tissue-specific regulation of allantoinase in *Phaseolus vulgaris L*. J Exp Bot.

[31] Berthelot K, Lecomte S, Estevez Y, Peruch F (2014). *Hevea brasiliensis* REF (Hev b 1) and SRPP (Hev b 3): An overview on rubber particle proteins. Biochimie.

[32] Luscher, M. *et al*. Cloning and functional analysis of sucrose: sucrose 1-fructosyltransferase from tall fescue. *Plan Physiol* **124**, 1217–1227 (2000). 10.1104/pp.124.3.1217 PMC59220 11080298

[33] Van Laere A, Van den Ende W (2002). Inulin metabolism in dicots: chicory as a model system. Plant Cell Environ.

[34] Van den Ende W, Michiels A, De Roover J, Van Laere A (2002). Fructan biosynthetic and breakdown enzymes in dicots evolved from different invertases. Expression of fructan genes throughout chicory development. Scientific World J.

[35] Ko., J. H., Chow., K. S., & Han., K. H. Transcriptome analysis reveals novel features of the molecular events occurring in the laticifers of *Hevea brasiliensis* (para rubber tree). *Plant Mol Biol* **53**, 479–492 (2003). 10.1023/B:PLAN.0000019119.66643.5d 15010614

[36] Priya P, Venkatachalam P, Thulaseedharan A (2007). Differential expression pattern of rubber elongation factor (REF) mRNA transcripts from high and low yielding clones of rubber tree (*Hevea brasiliensis* Muell. Arg.). Plant Cell Rep.

[37] Ponciano, G. *et al*. Transcriptome and gene expression analysis in cold-acclimated guayule (*Parthenium argentatum*) rubber-producing tissue. *Phytochemistry* **79**, 57–66 (2012). 10.1016/j.phytochem.2012.04.007 22608127

[38] Oelmuller R, Herrmann RG, Pakrasi HB (1996). Molecular studies of CtpA, the carboxyl-terminal processing protease for the D1 protein of the photosystem II reaction center in higher plants. J. Biol. Chem..

[39] Cornish K (2016). Temporal diversity of *Taraxacum kok-saghyz* plants reveals high rubber yield phenotypes. Biodiversitas.

[40] Cornish K, Backhaus RA (2003). Induction of rubber transferase activity in guayule (*Parthenium argentatum Gray*) by low temperatures. Ind Crops Prod..

[41] Goncalves PDS, Silva MD, Gouvea LRL, Scaloppi EJ (2006). Genetic variability for girth growth and rubber yield in *Hevea brasiliensis*. Sci. Agric..

[42] Hirsch S, Oldroyd GE (2009). GRAS-domain transcription factors that regulate plant development. Plant Signal Behav.

[43] Xue, L. *et al*. Network of GRAS transcription factors involved in the control of arbuscule development in *Lotus japonicus*. *Plant Physiol*. **167**, 854–871(2015). 10.1104/pp.114.255430 PMC4348782 25560877

[44] Boyes DC, Nam J, Dangl JL (1998). The *Arabidopsis thaliana* RPM1 disease resistance gene product is a peripheral plasma membrane protein that is degraded coincident with the hypersensitive response. Proc. Natl. Acad. Sci. USA.

[45] Stermer, B.A., Bianchini, G.M. & Korth, K.L. Regulation of HMG-CoA reductase-activity in plants. *J. Lipid Res.* **35**, 1133–1140 (1994). 7964176

[46] Rahman, A. Y. *et al.* Draft genome sequence of the rubber tree *Hevea brasiliensis*. *BMC Genomics* **14**, 75 (2013). 10.1186/1471-2164-14-75 PMC3575267 23375136

[47] Laranjeira, S. *et al*. Arabidopsis squalene epoxidase 3 (SQE3) complements SQE1 and is important for embryo development and bulk squalene epoxidase activity. *Mol Plant* **8**, 1090–1102 (2015). 10.1016/j.molp.2015.02.007 25707755

[48] Liu A, Burke JM (2006). Patterns of nucleotide diversity in wild and cultivated sunflower. Genetics.

[49] Gupta., P. K., Roy., J. K., & Prasad., M. Single nucleotide polymorphisms: A new paradigm for molecular marker technology and DNA polymorphism detection with emphasis on their use in plants. *Curr Sci* **80**, 524–535 (2001).

[50] Chapman MA, Burke JM (2007). DNA sequence diversity and the origin of cultivated safflower (*Carthamus tinctorius L.;* Asteraceae). BMC Plant Biol.

[51] Frazer KA, Elnitski L, Church DM, Dubchak I, Hardison RC (2003). Cross-species sequence comparisons: A review of methods and available resources. Genome Res..

[52] Hellier BC (2011). Collecting in Central Asia and the Caucasus: US National Plant Germplasm System Plant Explorations. HortScience.

[53] van Beilen JB, Poirier Y (2007). Establishment of new crops for the production of natural rubber. Trends Biotechnol.

[54] Li, B. & Dewey, C. N. RSEM: accurate transcript quantification from RNA-Seq data with or without a reference genome. *BMC Bioinformatics* **12** (2011). 10.1186/1471-2105-12-323 PMC3163565 21816040

[55] Ohio Supercomputer Center. (1987).

[56] Conesa, A. *et al*. Blast2GO: a universal tool for annotation, visualization and analysis in functional genomics research. *Bioinformatics* **21**, 3674–6 (2005). 10.1093/bioinformatics/bti610 16081474

[57] Huang X, Madan A (1999). CAP3: A DNA sequence assembly program. Genome Res.

[58] Leng N (2013). EBSeq: an empirical Bayes hierarchical model for inference in RNA-seq experiments. Bioinformatics.

[59] R Development Core Team. R: A Language and Environment for Statistics Computing. Vienna, Austria: R Foundation for Statistical Computing. Retrieved from http://www.R-project.org (2010).

[60] Suzuki R, Shimodaira H (2006). Pvclust: an R package for assessing the uncertainty in hierarchical clustering. Bioinformatics.

[61] Langmead B, Salzberg SL (2012). Fast gapped-read alignment with Bowtie 2. Nature Methods.

