# Supplementary material for: Analysis of the first Taraxacum kok-saghyz transcriptome reveals potential rubber yield related SNPs
Source: Sci Rep. 2017 Aug 30;7:9939. doi: 10.1038/s41598-017-09034-2 (PMC5577190; doi:10.1038/s41598-017-09034-2)
Supplement: Supplementary file 1 — Supplementary Figures [file 41598_2017_9034_MOESM1_ESM.doc]

**Analysis of the first *Taraxacum kok-saghyz* transcriptome reveals potential rubber yield-related SNPs**

Zinan Luo^Co^, Brian J. Iaffaldano^Co^, Xiaofeng Zhuang, Jonathan Fresnedo-Ramírez, Katrina Cornish*

**Contact Information**

Zinan Luo^Co^, [luo.356@osu.edu](mailto:luo.356@osu.edu), The Ohio State University, Department of Horticulture and Crop Science, Wooster OH, 44691, USA

Brian J. Iaffaldano^Co^, [iaffaldano.1@osu.edu](mailto:iaffaldano.1@osu.edu), The Ohio State University, Department of Horticulture and Crop Science, Wooster OH, 44691, USA

Xiaofeng Zhuang, [zhuang.97@osu.edu](mailto:zhuang.97@osu.edu), The Ohio State University, Department of Horticulture and Crop Science, Wooster OH, 44691, USA

Jonathan Fresnedo-Ramírez, [fresnedoramirez.1@osu.edu](mailto:fresnedoramirez.1@osu.edu), The Ohio State University, Department of Horticulture and Crop Science, Wooster OH, 44691, USA

Katrina Cornish*, [cornish.19@osu.edu](mailto:cornish.19@osu.edu), The Ohio State University, Department of Horticulture and Crop Science, Wooster OH, 44691, USA

**Supplementary Figures**


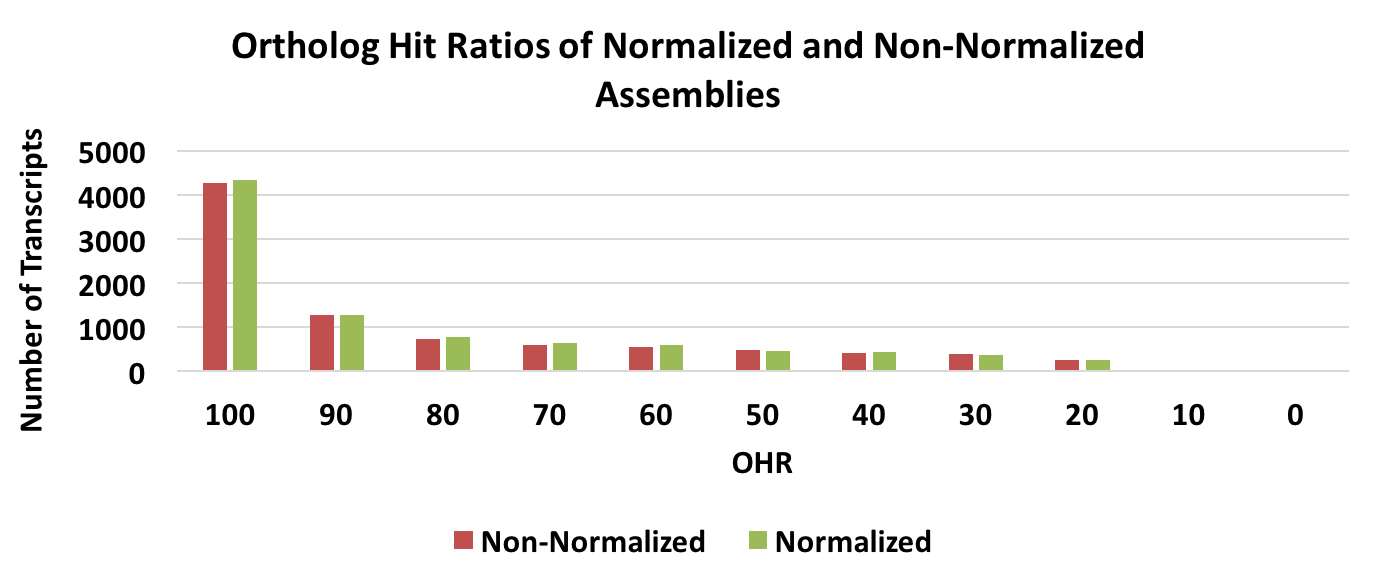


**Figure S1. The comparison of ortholog hit ratios (OHR) between normalized and non-normalized assemblies.**


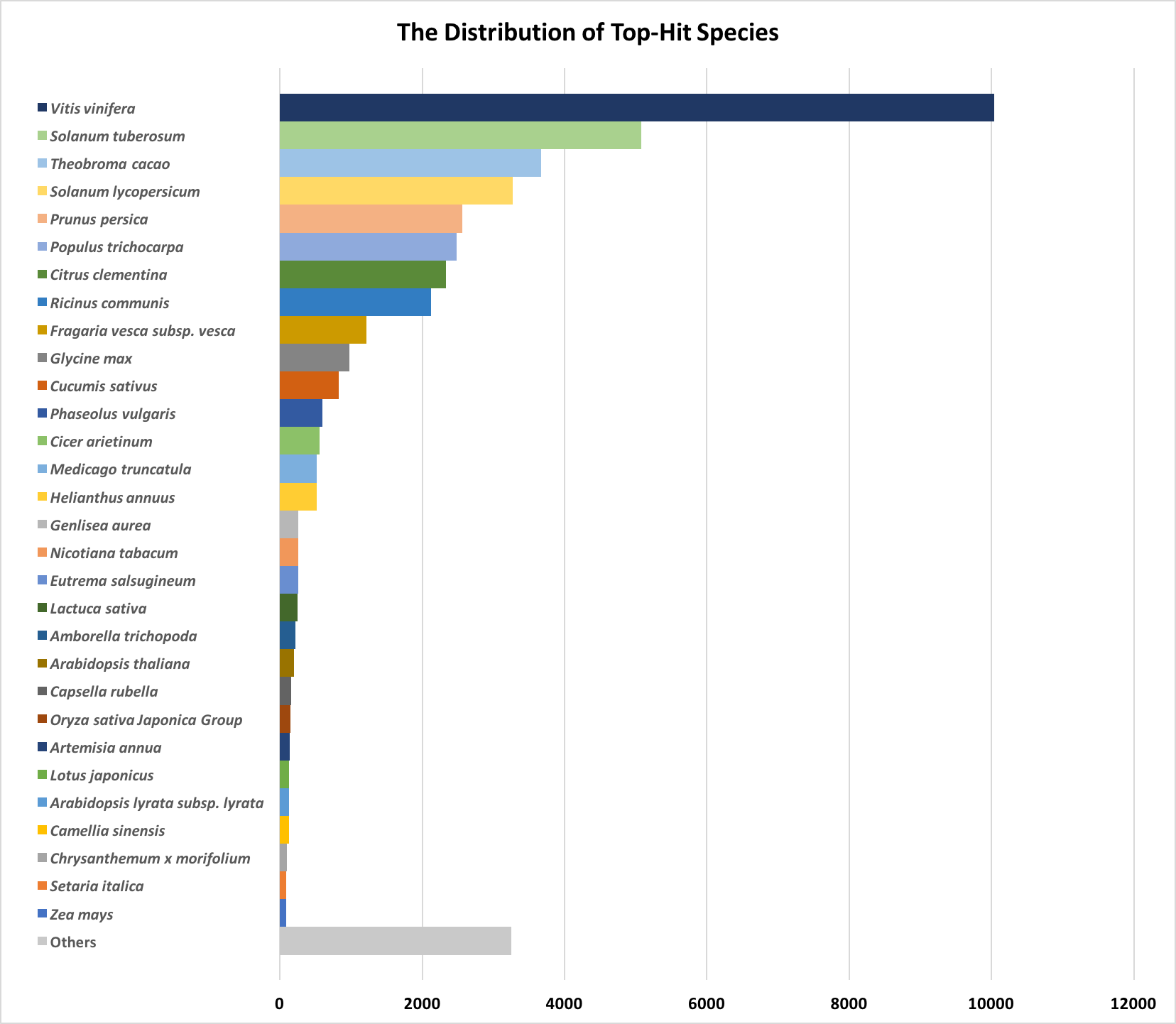


**Figure S2. Top-hit species distribution for RNA-Seq data**

**
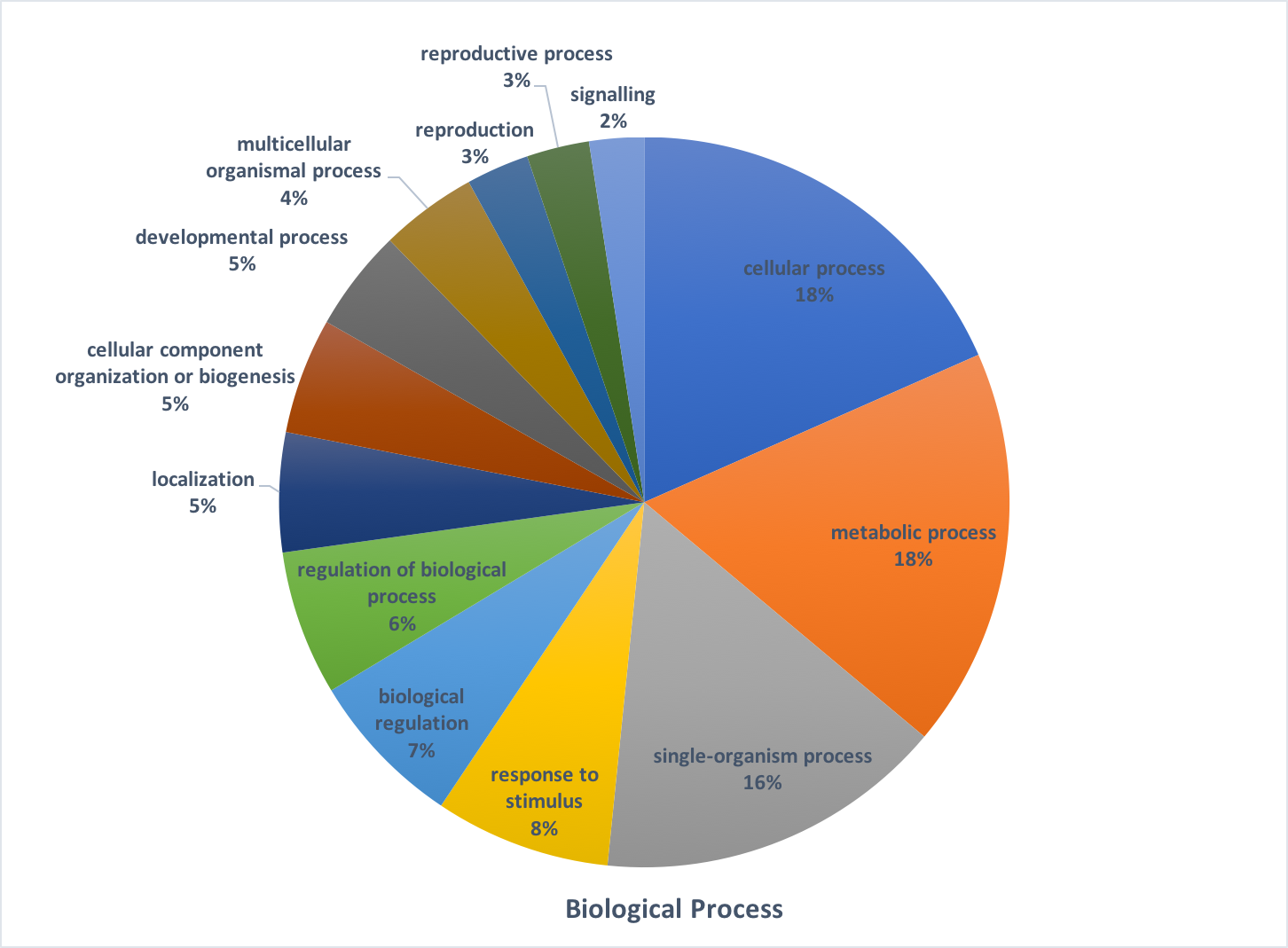
**

**
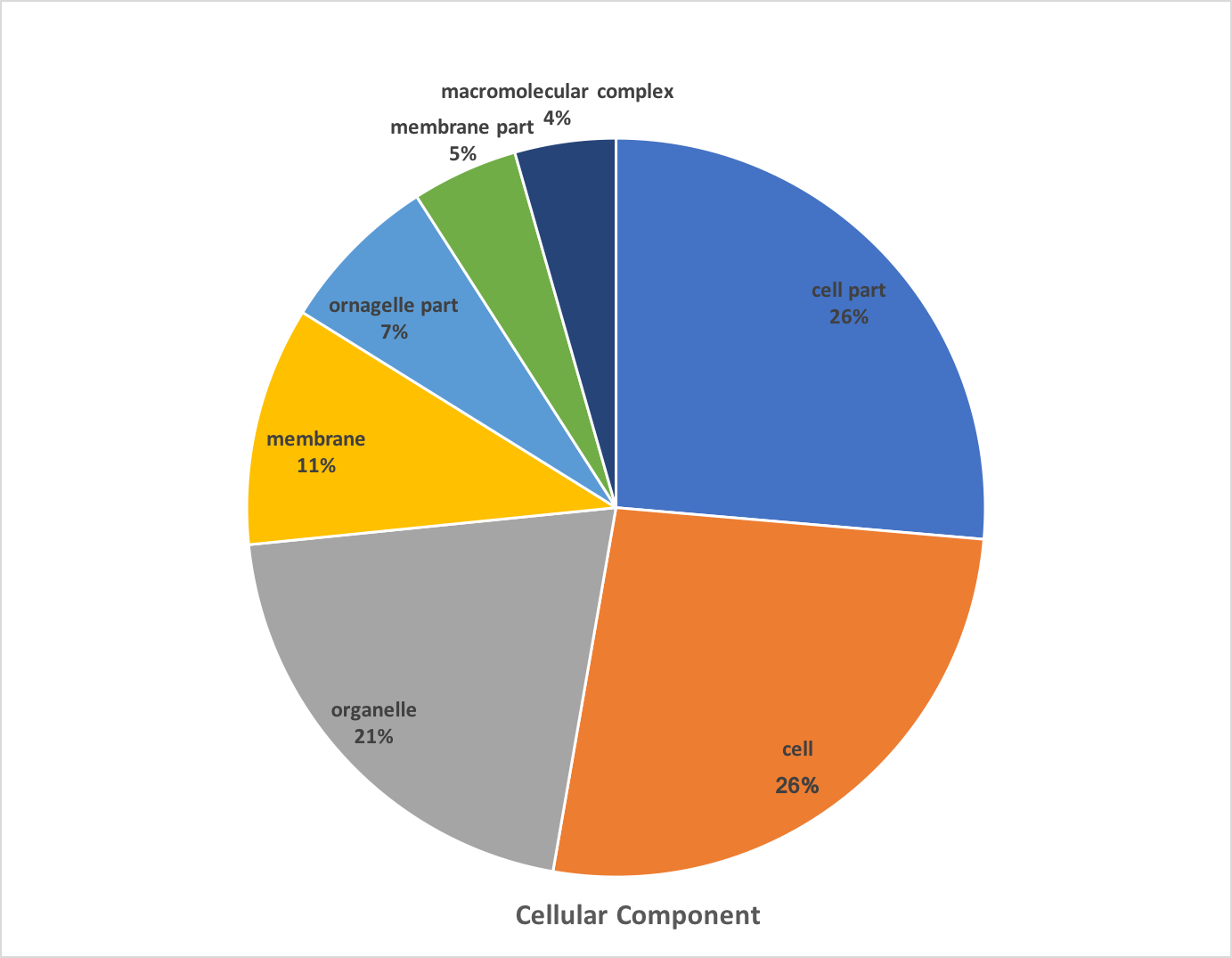

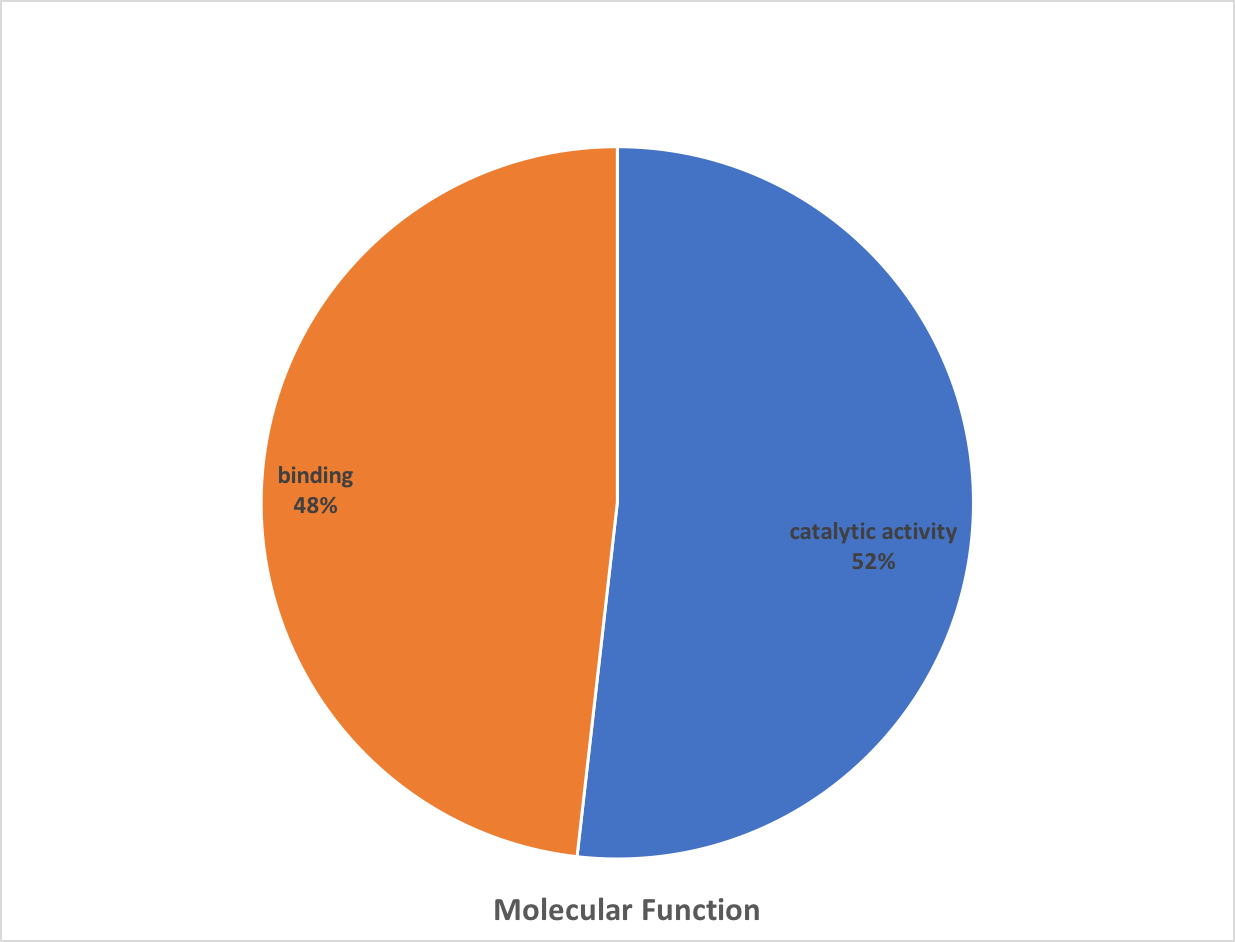
**

**Figure S3. GO terms analysis using Blast2GO under three categories on level 2: biological process, cellular component and molecular function.**

**
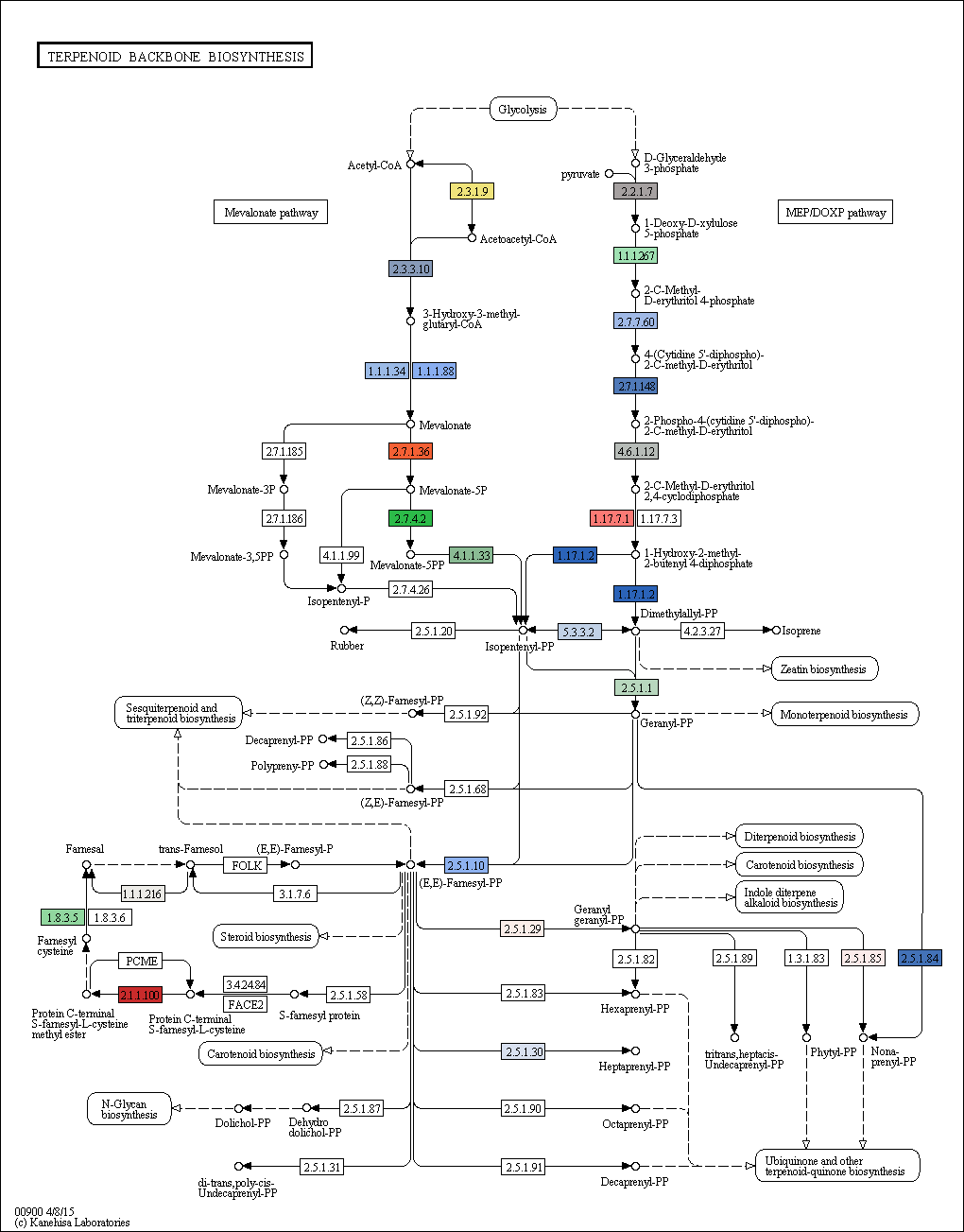
**

**Figure S4. The enzymes involved in the terpenoid backbone biosynthesis pathway^1^.** The colored ones are assigned by our transcripts.


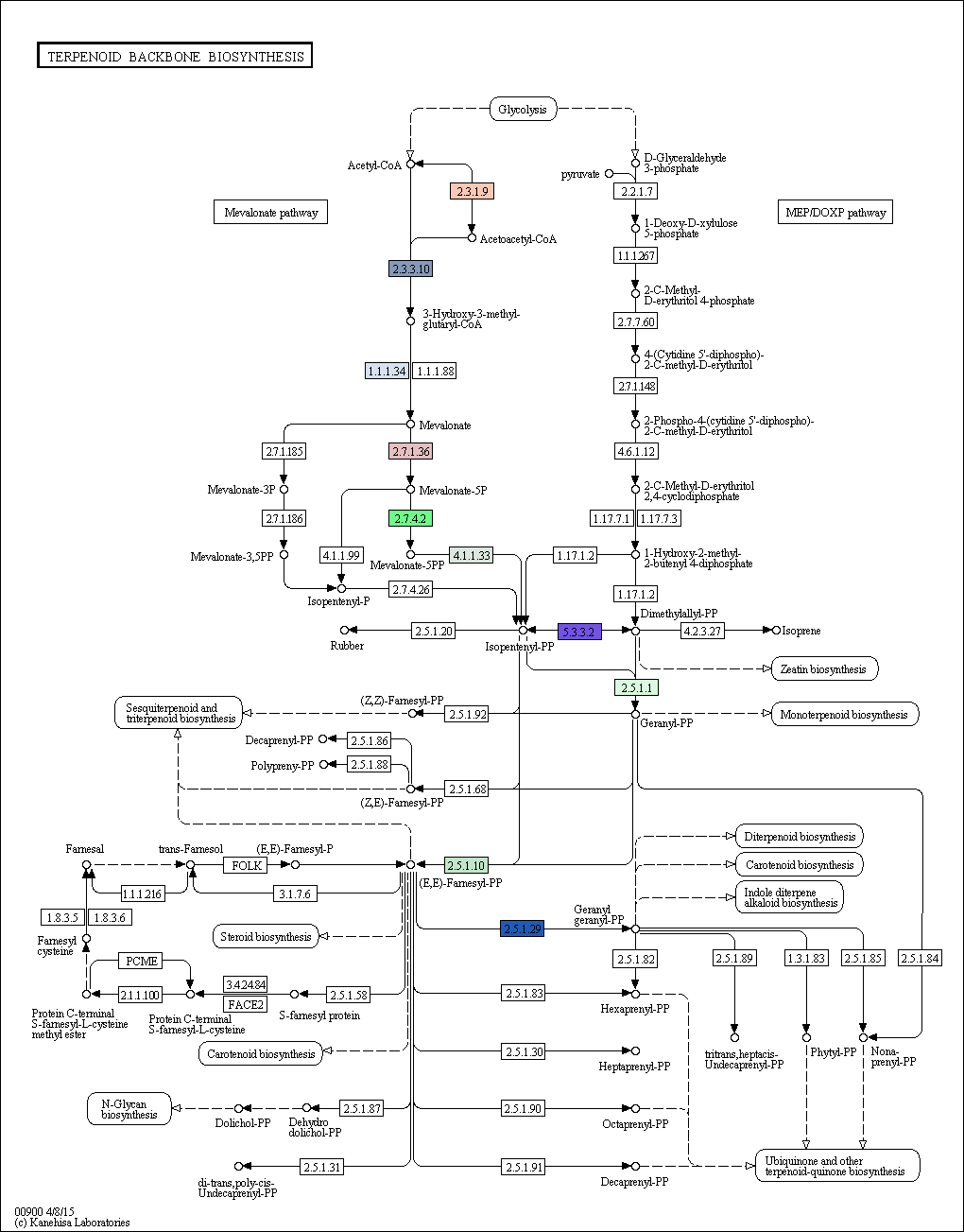


**Figure S5.** **The enzymes involved in the terpenoid backbone biosynthesis pathway^1^.** The colored ones are assigned by online TK root ESTs.

**Reference**

1. Kanehisa, M., Furumichi, M., Tanabe, M., Sato, Y. & Morishima, K. KEGG: new perspectives on genomes, pathways, diseases and drugs*. Nucleic Acids R*e**s** 45, D353-D361 (2017).
